# Effect of Chemical Challenges on the Properties of Composite Resins

**DOI:** 10.1155/2021/4895846

**Published:** 2021-12-01

**Authors:** Omar Geha, Luciana Tiemi Inagaki, Jaqueline Costa Favaro, Alejandra Hortencia Miranda González, Ricardo Danil Guiraldo, Murilo Baena Lopes, Sandrine Bittencourt Berger

**Affiliations:** ^1^Department of Restorative Dentistry, School of Dentistry, University North of Parana, Londrina, Paraná, Brazil; ^2^State University of Londrina, Department of Oral Medicine and Pediatric Dentistry, Londrina, Paraná, Brazil

## Abstract

**Objective:**

To evaluate the chemical degradation effect on microhardness and roughness of composite resins after aging.

**Materials and Methods:**

Specimens (*n* = 10) were used for Filtek Z350 XT (Z350), Filtek Bulk Fill (BULK), Micerium HRI (HRI), Micerium BIOFUNCION (BIO), and Vittra APS (VITTRA). Microhardness and roughness were performed before and after degradation with the followed solutions: citric acid, phosphoric acid, 75% alcohol, and distilled water. Samples were to a 180-day chemical cycling protocol. After degradation, one sample of each group was selected for scanning electron microscope evaluation. The data were analyzed with normal distribution (Kolmogorov–Smirnov) and similarities of variations for the Bartlett test. ANOVA (two-way) followed by Tukey's test was performed considering treatment and composite resin (*P* < 0.05).

**Results:**

For microhardness and roughness, variations were noted to different solution and resin formulations. Z350 and HRI showed higher microhardness percentage loss, and it was more evident after storage in alcohol (−48.49 ± 20.16 and −25.02 ± 14.04, respectively) and citric acid (−65.05 ± 28.97 and 16.12 ± 8.35, respectively). For roughness, Z350 and VITTRA showed less delta values after alcohol storage (−0.047 ± 0.007 and −0.022 ± 0.009, respectively). HRI had the worst roughness for citric acid (−0.090 ± 0.025). All resins were not statistically different between each other in water and phosphoric acid.

**Conclusion:**

The formulations of restorative resin materials influenced in degree of surface degradation after 180 days of chemical degradation. Water was considered the solution that causes less degradation for microhardness and roughness evaluations. For microhardness, alcohol was considered the worst solution for Z350 and HRI. For superficial roughness, Z350 and VITTRA showed less degradation in alcohol and citric and phosphoric acid solutions.

## 1. Introduction

Since the composite resins have been developed, these materials have undergone many changes in composition and properties with the intention to improve dental restorations durability [1]. In this context, the presence of filler particles has become a determining factor in the mechanical behavior of polymer-based restorations, improving abrasion resistance, increasing mechanical strength, and reducing shrinkage and polymerization stress [2–4]. The filler particles' size can also affect surface smoothness and susceptibility to extrinsic staining and external discoloration such as hygiene, diet, and smoking habits [5–7]. So, with the increase in demand for extremely esthetic direct restorative materials and the necessity to improve physical and mechanical properties, the introduction of nanotechnology in composite resins was growing up [8, 9].

Modified resins as bulk fill were presented to the dentistry community proposing the use of increments of restorative material up to 4 mm in thickness, presenting less contraction of volumetric polymerization, resulting in low tension of contraction [10–13]. In addition, with intension to make the nanoparticle composite resins closer to natural teeth tissue characteristics, health biocompatible new formulations of materials have been developed with free comonomers of BisGMA and nanofilling glass that provide similar characteristics of natural enamel, improving the restoration esthetic aspect and durability [14, 15]. Another way to reach better performance of composite resin restorative materials is to work modifying the polymerization systems of materials as the resins with an advanced polymerization system (APS) [16]. This recent technology was developed to increase important characteristics of composite resins: to guarantee the high percentage of degree of conversion, increase bond strength, and provide high quality of esthetic profile [14–16].

In view of the mechanical and chemical challenges present in the oral cavity, such as variations in temperature, acidity, and contact with pigmenting food agents, there is a compromise in color stability and degradation of the restoration, with the appearance of undesirable effects, taking into account that the consumption of acidic drinks by the population has increased a lot in the last years, and it is necessary to identify if the acids commonly present in these drinks cause the degradation of the resin matrix [6, 7, 12, 17]. Furthermore, other effects such as the alteration of surface roughness and the decreased microhardness on composite resins are also reported [18].

Thus, in front of the new formulations of composite resins available and understanding the imminent staining process that all materials can suffer into the oral cavity, this study aimed to evaluate the effect on microhardness and roughness properties of composite resins with different compositions after chemical degradation. The hypothesis tested was that different formulations of composite resins could interfere in degree of chemical degradation.

## 2. Materials and Methods

### 2.1. Specimen Preparation

In this study, five groups of commercial restorative composite resins were used (Table 1): Filtek Z350 XT (3M ESPE, St. Paul, MN, USA); Filtek Bulk Fill (3M ESPE, St. Paul, MN, USA); Micerium HRI (MICERIUM S.p.A., Avegno, GE, Italy); Micerium BIOFUNCION (MICERIUM S.p.A., Avegno, GE, Italy); and Vittra APS (FGM, Joinville, SC, Brazil). Cylindrical specimens (*n* = 10) were made using a metallic mold (6.0 ± 0.1 mm of diameter for 0.8 ± 0.1 mm of thickness), according to ISO 4049 recommendations [19]. A polyester strip covered with a glass plate was placed over the top of mold, and a force of 50 g was used for 30 seconds to obtain a smooth and standard flat surface [20]. After this, each specimen was light cured for 40 seconds with the LED Radii-Cal (SDI, Baywater, Victoria, Australia) device with an irradiance of 1200 mW/cm^2^ and stored in 100% humidity at room temperature for 24 h at 37°C for microhardness and roughness initial evaluation.

### 2.2. Superficial Microhardness Evaluation

The microhardness was evaluated before and after chemical degradation. So, for Vickers microhardness readings, a microdurometer (HMV-G 21S, Shimadzu Corporation, Japan) was used with a load of 50 wtg for 5 seconds. All evaluations were performed on the top, in the center of the specimen, and 3 indentations were performed with 100 *μ*m of distance with each other. The final microhardness value was obtained for the average of indentations, and a percentage of microhardness loss was considered as final data.

### 2.3. Roughness Evaluation

The roughness evaluation was performed after microhardness evaluation, before and after chemical degradation. A digital profilometer (SJ-410, Mitutoyo, Japan) was used to sweep the surface with 0.25 mm of extension in three different directions. The final value of superficial roughness (Ra, *μ*m) was obtained for Ra average, and a delta value was calculated for final data (Δ_R_ = inicial_R_ − final_R_).

### 2.4. Chemical Degradation

For chemical degradation, 4 different solutions were prepared: citric acid 0.02 N (pH < 6.0), phosphoric acid 0.02 N (pH < 7.0), 75% alcohol, and distilled water (control group) [20]. The samples were stored, separately, in 5 mL of distilled water at 37°C. For the chemical degradation, a cycle protocol of one minute immersion in 5 mL of each solution, 4 times per day at 37°C for 180 days was followed [17]. After each chemical challenge, all samples were kept stored in distilled water at 37°C.

### 2.5. Scanning Electron Microscope Evaluation

**Figure 1:**
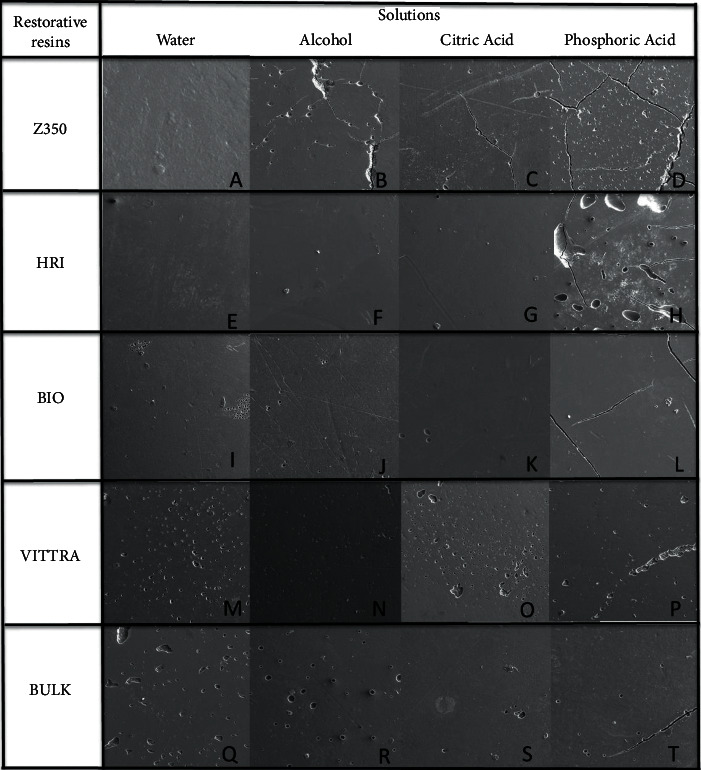
SEM photomicrographs of resin surface after 180 days of chemical degradation.

The scanning electron microscope (SEM) was used for superficial surface of one sample for each group of resins after chemical degradation. The magnification of 200× was used to acquire composite resin surface images (Figure 1).

### 2.6. Statistical Analysis

The statistical analysis was performed using Minitab 16 for Windows 8 software (Minitab Inc., Pennsylvania State College, Philadelphia, PA, USA). The data were analyzed with normal distribution using the Kolmogorov–Smirnov test. The similarities of variations were evaluated for the Bartlett test. After checking the normal distribution of data, ANOVA (two-way) followed by Tukey's test was performed considering the following factors: treatment (water, alcohol, citric acid, and phosphoric acid) and composite resin (BIO, BULK, HRI, VITTRA, and Z350). A 5% significance level was considered for this analysis.

## 3. Results

**Figure 2:**
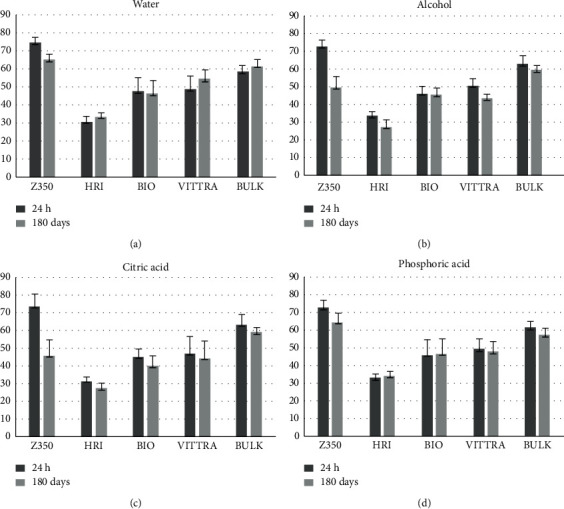
Materials' microhardness (error bars: standard deviation) surface after 24 hours and 180 days of chemical solutions degradation.

In this study, the Vickers microhardness of composite resins tested had variations in relation to loss and gain of hardness when they are stored in water, alcohol, citric acid, and phosphoric acid (Table 2). After 180 days of degradation, comparing each composite, Z350 and HRI showed higher alterations of microhardness than the others composite resins, and it was more evident after storage in alcohol and citric acid than in water and phosphoric acid. However, HRI also presents increase of microhardness after storage in water and phosphoric acid. BIO and BULK presented similar behavior in all solutions, and they were not statistically different between each other. VITTRA when stored in water showed less change than the values, and it was similar to that in phosphoric acid. The comparison between initial and final microhardness values in all solutions is shown in Figure 2.

**Figure 3:**
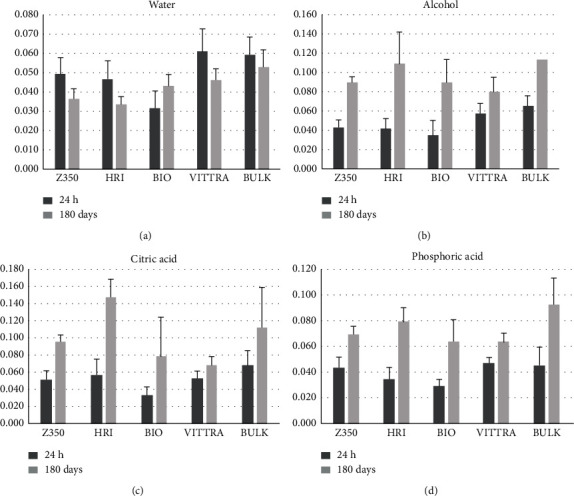
Materials surface roughness (error bars: standard deviation) after 24 hours and 180 days of chemical solution degradation.

Concerning roughness, all composite resins did not show statistical difference between each other after water storage, presenting a tendency to be less rough after degradation. Nevertheless, for other solutions, the composite resins presented roughness increase after the degradation period (Table 3). VITTRA showed less rough surface than HRI, BIO, and BULK after alcohol storage, and it was not statistically different than Z350. HRI had the worst roughness surface than the other groups of resins for citric acid. For phosphoric acid, all resins were not statistically different between each other. The comparison between initial and final roughness values in all solutions is shown in Figure 3.

In relation of SEM images obtained after degradation, Z350 showed higher alterations in microhardness and roughness surface in alcohol solution after chemical degradation challenge. The SEM shows that water had the best performance after 180 days. In alcohol and citric and phosphoric acid, we can observe the presence of evidenced fissures in the images (Figure 1B, 1C, and 1D). HRI presented bubble formation, and it can be observed when the samples were treated with phosphoric acid (Figure 1H), for other solution, no alterations were observed (Figure 1E, 1F, and 1G). BIO showed no significant changes in water (Figure 1I), alcohol (Figure 1J), and citric acid (Figure 1K). Cracks are observed when the samples were immersed in phosphoric acid (Figure 1L). For VITTRA, the treatment with water (Figure 1M), alcohol (Figure 1N), and citric acid (Figure 1O) did not promote significant surface changes. However, samples submitted to phosphoric acid showed higher alterations (Figure 1P). Regarding BULK, the SEM showed similar changes in water (Figure 1Q), alcohol (Figure 1R), citric acid (Figure 1S), and phosphoric acid (Figure 1T).

## 4. Discussion

The restorative materials, including resin composites, have been exposed to severe conditions such as chemical challenges in the oral cavity. In acid environments, resin composites can suffer loss of important properties as decrease of microhardness and increase of surface roughness, becoming a weakness for the quality of restorations [17, 21]. In this context, with the intention of improving the resistance of resin materials, new materials are being developed and disposable in the market [14–16]. The resin materials used in this study are widely used for restoration treatments nowadays, with nanoproperties technology, BisGMA-free, and new photoinitiators agents; furthermore, few studies have been conducted verifying materials' resistance performance [16, 22]. Like this, in this study, the resins analyzed showed changes in microhardness and roughness properties after degradation in water, alcohol, citric acid, and phosphoric acid solutions for 180 days of chemical challenge, and the hypothesis could be accepted as soon as composition of each resin determined the degradation.

In relation to the chemical degradation protocol [17], the solutions simulated drinks consumed for all people around the world as citric juices, alcoholic drinks, and acid phosphoric present in soft drinks. It is known that acidic environments could produce high levels of alterations in resin materials surfaces [23–25] and with the intention to evaluated extreme conditions of degradation, the specimens were submitted to chemical degradation in interrupted 180 days. Ethanol is frequently used to investigate particles' elution from composites because it is an organic solvent with capacity to penetrate the polymeric matrix [26]. The ethanol effect could be investigated according to degree of elution and composition of materials, mainly in relation to the organic matrix of resin materials [26, 27]. In the same line, the citric acid also is considered an organic acid with high levels of dental/restorations erosion capacity [27]. In this study, the SEM images after chemical challenge showed more traces of degradation as depressions, bobbles, and cracks in alcohol and citric and phosphoric acid solutions than in water (Figure 1), probably for high capacity of dilution of these acid solutions. This fact could suggest that an environment with low pH could affect resin materials' surfaces, damaging properties as microhardness and roughness [28].

Concerning microhardness, after storage of 180 days in chemical solutions, the resin Z350 suffered more variations in relation to microhardness percentage in all environments tested, losing hardness properties in all solutions. This fact could be associated with the presence of highly soluble monomers in resin composition as BisEMA, TEGDMA, UDMA, and BisGMA in resin composition [29]. BisEMA, TEGDMA, and UDMA are dymetacrilate monomers recognized for their high fluid properties and their high performance of degree of conversion, giving better quality of polymerization [4, 29]. As BisGMA is a very viscous monomer, BisEMA, TEGDMA, and UDMA are usually used as diluent monomers for increasing BisGMA flexibility in the polymerization process [29]. Nevertheless, BisGMA, BisEMA, TEGDMA, and UDMA are very soluble monomers [29], and this fact could be influenced in Z350 worst performance after chemical degradation in comparison to other resins tested. BIO, BULK, HRI, and VITTRA presented similar behavior in solutions, except for alcohol solution where BULK and BIO presented less loss of microhardness followed by VITTRA. The presence of inorganic particles as zinc, silica, barium, or zirconia in the organic matrix [22] could improve the resistance of resin materials for degradation challenges, and this fact could be influenced by the high performance of resin composites as BIO, BULK, and VITTRA.

When surface roughness was evaluated, VITTRA and Z350 showed the best performance in all acid solutions tested, with low roughness variations. These results could also be associated with VITTRA and Z350 composition, discussed above for microhardness evaluation. Both resins showed more resistance for degradation because of the presence of monomer diluents. TEGDMA and UDMA dymetacrilates can guarantee high percentage of polymerization that contribute for more condensed polymeric matrix and, consequently, contribute for a resistant smooth surface [29–31]. On the other hand, dymetacrilate monomers are very soluble [29, 32] and in high percentage in the composition can affect properties as hardness that could be noted in this study with Z350. In this case, VITTRA showed advantage because of the presence of nanoparticles of silica and zirconia that contributed for more resistance to chemical challenge.

It is important to note that all experiments in this study were accomplished *in vitro*, and although rigorous protocols have been followed, some limitations to simulate oral cavity environmental challenges still could be present. Nevertheless, important results were found about VITTRA about chemical degradation resistance. The fact that VITTRA has an advantageous polymerization system with combination of different photoinitiators [16] could be interfered in high performance of polymeric matrix formation that contributed for good results after degradation. Another point that could be considered is about VITTRA composition, with high percentage of chemical elements than other tested resins, as carbon, silica, and aluminum that could be reinforced by the VITTRA performance [22].

So, in front of the data obtained in this study, VITTRA could be considered the material that showed the best results for chemical degradation. If a rank, in this study, would be made for resin materials' resistance after chemical challenge, the followed suggestion could be considered: VITTRA > Z350 > BULK > BIO > HRI. Clinical studies could be performed to complement the data obtained concerning chemical resistance from composite resins investigated in this study.

## 5. Conclusions

According to the tests evaluated in this study, the formulations of restorative resin materials influenced surface degradation after immersion in different chemical solutions for 180 days. For microhardness, the challenge in water caused less degradation for all materials, and alcohol was considered the worst solution, mainly for Z350 and HRI. For superficial roughness, water also was considered the solution that caused less degradation for all materials, and Z350 and VITTRA were the materials that showed less degradation in alcohol and citric and phosphoric acid solutions.

## Figures and Tables

**Table 1:** Commercial restorative composite resins data, according to the manufacturer's information.

Composite resin	Classification	Composition/filler
Filtek Z350 XT (Z350)	Conventional nanoparticulate	BisGMA, UDMA, TEGDMA BISEMA, Zr, Si/75 wt% and 59.5 vol%
Micerium HRI (HRI)	Conventional nanohybrid	1,4-BUTANDIOLDIMETHACRILATE UDMA, BISGMA/80 wt% and 63 vol%
Micerium BIOFUNCTION (BIO)	Conventional nanohybrid	1,4-BUTANDIOLDIMETHADRILATE UDMA, BPA free/74% wt% and 60 vol%
Vittra APS (VITTRA)	Conventional nanoparticulate	UDMA, TEGDMA, photoinitiator composition (APS), Zr, Si, BPA free/72–82 wt% and 52–60 vol%
Filtek bulk fill (BULK)	Bulk fill nanohybrid	Ceramic treated with silane, UDMA, DDDMA, EDMAB, benzotriazol, TiO_2_/64.5 wt% and 42.5 vol%

**Table 2:** Percentage of Vickers microhardness alterations after 180 days of degradation.

Composite resins	Solution treatments			
	Water	Alcohol	Citric acid	Phosphoric acid
Z350	−15.22(6.50)^Ba^	−48.49(20.16)^Cb^	−65.05(28.97)^Bb^	−13.20(9.31)^Ba^
HRI	8.70(9.81)^ABa^	−25.02(14.04)^Cb^	−16.12(8.35)^Ab^	3.21(1.93)^Aa^
BIO	−2.21(1.54)^ABa^	−1.81(1.06)^Aa^	−12.45(7.59)^Aa^	1.52(0.84)^ABa^
VITTRA	9.77(9.83)^Aa^	−16.86(10.26)^Bb^	−8.45(8.24)^Ab^	−2.93(1.40)^ABab^
BULK	5.15(4.19)^ABa^	−5.81(4.18)^Aa^	−7.77(6.77)^Aa^	−6.64(4.24)^ABa^

Different capital letters (in column) and small letters (in line) are statistically different from each other for Tukey's test (*P* < 0.05).

**Table 3:** Delta values of roughness surface (Δ_R_ = initial_R_ − final_R_) after 180 days of degradation in different solutions.

Composite resin	Solutions treatments			
	Water	Alcohol	Citric acid	Phosphoric acid
Z350	0.013(0.008)^Aa^	−0.047(0.007)^ABb^	−0.044(0.011)^Ab^	−0,026(0.006)^Ab^
HRI	0.013(0.010)^Aa^	−0.067(0.031)^Bbc^	−0.090(0.025)^Bc^	−0,045(0.011)^Ab^
BIO	−0,012(0.007)^Aa^	−0.055(0.014)^Bb^	−0.046(0.040)^Ab^	−0,035(0.015)^Aab^
VITTRA	0.015(0.011)^Aa^	−0.022(0.009)^Ab^	−0.016(0.011)^Aab^	−0,016(0.007)^Aab^
BULK	0.006(0.001)^Aa^	−0.048(0.032)^Bb^	−0.045(0.041)Ab	−0,047(0.016)^Ab^

Different capital letters (in column) and small letters (in line) are statistically different from each other for Tukey's test (*P* < 0.05).

## Data Availability

The data used to support the findings of this study are available from the corresponding author upon request.
